# Scenario planning for community development in Vietnam: a new tool for integrated health approaches?

**DOI:** 10.3402/gha.v7.24482

**Published:** 2014-08-18

**Authors:** Vi Nguyen, Hung Nguyen-Viet, Phuc Pham-Duc, Martin Wiese

**Affiliations:** 1 Centre for Public Health and Ecosystem Research, Hanoi School of Public Health, Hanoi, Vietnam; 2 Department of Epidemiology and Public Health, Swiss Tropical and Public Health Institute, Socinstrasse, Basel, Switzerland, and Food Safety and Zoonoses Program, International Livestock Research Institute, Hanoi, Vietnam; 3 Swiss Federal Institute of Aquatic Science and Technology, Sandec – Department of Water and Sanitation in Developing Countries, Dübendorf, Switzerland; 4 Infrastructure Department, Embassy of the European Union in Chad/European Union Delegation in Chad, N'Djamena, Chad

**Keywords:** integrated approaches, tools, scenarios, planning, sanitation, development, Vietnam

## Abstract

**Background:**

Like many countries in Southeast Asia, Vietnam's rapid population and economic growth has met challenges in infrastructure development, especially sanitation in rural areas.

**Objective:**

As an entry point, we developed scenario planning as an action–research tool in a peri-urban community to identify first steps towards improving their complex sanitation problem and to, systemically, address emerging/re-emerging infectious diseases, as these are commonly linked to unsafe water and inadequate sanitation conditions. As an integrated approach, the process of constructing scenarios allowed us to work across sectors and stakeholders to incorporate this knowledge into a common vision.

**Design:**

We conducted focus group discussions to identify and rank driving forces, orally constructed scenarios for the most uncertain drivers, discussed scenario implications and options, and examined the overall process for usefulness and sustainability. During a one-month scoping phase and in between focus group meetings, we carried out household visits which helped us understand the context of data and gather feedback from participants outside of the formal data collection process. Recorded results from these activities were used to develop subsequent tools.

**Results and Conclusions:**

The research process gave us insights into how to adapt the scenario planning tool to identify alternative options. This involved choosing boundary partners, negotiating priorities, drawing out participant learning through self-assessment of our process (a prerequisite for changing mental models and thus achieving outcomes), and understanding how conveyed messages may reinforce the status quo. These insights showed the importance of examining research results beyond outputs and outcomes, namely through process.

Globally, official statistics estimate that 2.5 billion people lack access to ‘improved sanitation’ (1). Among associated health impacts are diarrhoeal diseases, which are the second leading contributor to global disease burden – greater than the combined impact of HIV/AIDs, malaria, and tuberculosis for children under five (2). Most of those in this sanitation gap live in Asia, where populations and economies have been growing rapidly (1). Despite progress in sanitation coverage, there are still significant barriers to addressing both land and finance requirements and the health and environmental impacts of waste management (3, 4).

Current approaches have focused on institutional transformation, household behaviours and community action, creating demand for sanitation, increased range of technologies and choices, and improving the funding mechanisms in sanitation and hygiene promotion (3). While important, many interventions, such as latrine construction and proper disposal of human excreta, require behavioural change or funding and resources that communities find very difficult to sustain beyond project funding cycles. Calls for integration and coordination in addressing issues that span the health and environment sectors have emphasized systems approaches, while cognizant of equity and meaningful participation of vulnerable populations (3, 5–8). This raises the question of how this can be done practically within communities.

## Scenario concept

Scenario planning allows researchers to work across sectors and stakeholders and incorporate the knowledge gained into a common vision (9). Scenarios are stories about possible futures and can provide stakeholders with a range of options for action on a shared issue (10). The heart of the process is having strategic conversations that focus on imminent change, which can be found throughout centuries and millennia (11). Molitor *et al*. has highlighted many predecessors of strategic conversations, which include operations research and systems analysis (methodologies in the context of decision-making); myths, legends, and tales (all types of stories); science fiction themes (inspiring deliberations of distant futures); brainstorming (the ‘free-association approach’); and lateral thinking (‘thinking outside the box’) (11). In the 18th century, scenarios were originally visionary and used for situations of uncertainty to help armies survive and win wars (12). Following the Second World War, scenario planning has been widely used for businesses in response to the failure of quantitative models (Table 1). Modern scenario development is credited to the futurist, Herman Kahn, based on techniques adapted from systems analysis and operations research (14).

**Table 1:** Scenario planning stages and activities for different contexts

Business context (13)	Community development context				

Steps	Focus group	Stages	Activities
Identifying focal issue Identifying key forces influencing issue in the local environment and their driving forces	1	Opening a time-perspective, connecting to experience	Refined focal issue Identified and ranked driving forces by importance
Ranking these by importance and uncertainty Selecting scenario logics	2	Analyze and project	‘Told’ scenarios for the most uncertain, ranked, paired combinations of drivers
Fleshing out scenarios Discussing their implications	3	Scale to real-life first steps	Listened to scenarios Discussed implications, options, and next steps
Selecting leading indicators for monitoring Adapting plans accordingly	4	Self-assessment of scenario process – build ownership	Discussed usefulness, sustainability, and application of scenarios building process/tool

Constructing stories is an iterative process of gathering information from participants, incorporating it into a scenario structure, and circulating for comments (15). Interview themes form the basis for constructing scenarios, which can be developed deductively, inductively, or normatively, according to Shell, one of the pioneers of contemporary scenario planning; other options are the techniques used to develop strategic conversations as cited above from Molitor *et al*. (11). In deductive scenarios, two critical uncertainties are chosen and represent the extremes on a matrix; storylines are developed for each quadrant. Inductive scenarios use a chain of events and a storyline is developed by describing how each unfolded. Normative scenarios begin with a set of characteristics at the end of the time horizon and are constructed backwards (15).

More recently, scenario planning has emerged in public health practice for departmental planning or national initiatives and in environmental sciences for informing investment decisions and improving ecological management (16–22). This has enabled community participation, where scenarios have been useful as a communication tool and a process to convene discussion on a shared issue (15). On one hand, businesses and organizations generally have the funding and infrastructure to support these activities and the power and resources to act on the decisions they identify, while in communities, the lack of resources and access to decision-making power are important limitations to scenario planning. Research that has used scenarios at the community level have been implemented by an external facilitator who initiates and directs the process, writes the scenarios based on interview data, and typically ends with a community presentation or trying to advocate for policy changes (20, 23, 24). Our research explored how to adapt scenario planning for action–research that could be owned by community members and highlighted opportunities and limitations of the process within our study context.

## Present investigation

### Study site

The research was conducted in Northern Vietnam, 60 km south of Hanoi, in Hoang Tay Commune, in the Kim Bang District of Hanam Province. As of March 2011, Hoang Tay had a population of 1,785 households, made up of 5,580 people (Le U, Personal communication, 2011). Situated within the Red River Delta, the commune has a sub-tropical climate with a rainy season from April to September and annual humidity varying from 80 to 90% (25). Agriculture and aquaculture were the major land uses and livelihoods, mainly rice and vegetable cultivation and poultry and pig farming. There were two main rice production cycles: January–June and July–October. At the time the research was carried out, the farmers were mainly women, while men worked in construction jobs or in factories in a nearby industrial park.

**Fig. 1:**
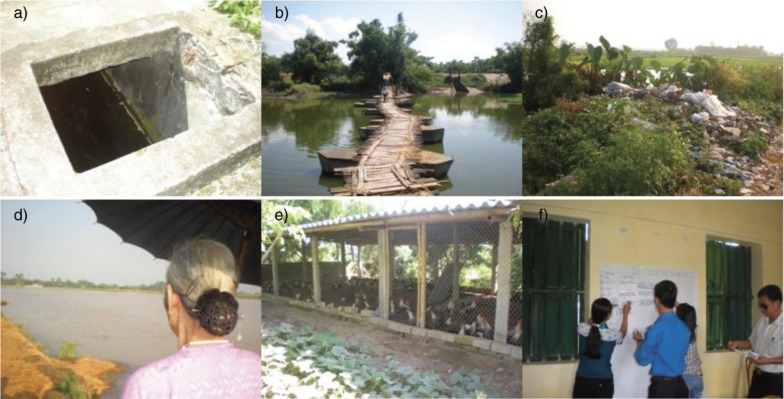
Photographs of (a) cistern, (b) bridge crossing the Nhue River, (c) roadside garbage, (d) community walk with an elder, (e) chicken pen during a household visit, and (f) focus group activities in Hoang Tay Commune, Kim Bang District, Hanam Province, Vietnam.

The local sanitation situation presented some public health risks to community members (Fig. 1). Diarrhoea cases were estimated at 814/100,000 people in 2008 (26, 27). Aggregated data for Hoang Tay and the neighbouring commune showed high prevalence of helminth infections. Exposure was apparently from unsanitary latrines and the use of human and animal excreta as fertilizer (both failing to meet national sanitary standards) and livestock raised adjacent to houses and water sources (28). Household water came from cisterns (boiled pre-consumption), while bathing and cleaning water was pumped from dug wells (26, 29). Further public health risk stemmed from the polluted Nhue River, the agricultural irrigation source, receiving untreated effluent from Hanoi (30).

Our study was linked to a partner programme with research activities that tested a conceptual framework for health and environmental sanitation in Vietnam (31, 32). In previous field work that examined their project for impediments and enablers of ecohealth practice, community capacity development was identified for further research efforts, which led to the present research study. Community members were discouraged by the lack of positive changes over the long-term and the scale of the problem (sewage flowing downstream from Hanoi and arsenic underground) and wished to get their children out of an inevitable undesirable future. This study aimed to test a scenario planning process to identify first steps towards improving the situation.

### Study approach: scoping participants and tools

The field work was conducted during July and August of 2011 and began with introductions to one village health worker in each of the 10 villages after we obtained permission from local leaders and ethics approval from the Hanoi School of Public Health (Decision No. 011–029/DD-YTCC) (Fig. 1). Each village health worker introduced us to three households in their village; one each, from the Women's Union, Farmer's Union, and Elder's Union (a total of 40 community members).

Eight community members from the People's Council participated. This was a strategic choice, mindful of the sustainability of scenario planning efforts, intended to take advantage of the existing infrastructure of Vietnam's mass organizations. Rather than choosing people from one particular mass organization, the People's Council was chosen due to anticipated opportunities for influence; it included leaders and would less likely be affected by changes in leadership since they represented different mass organizations. The scenarios were developed using focus group meetings with the same participants throughout the process. Initially, we tested community walks with two elders, community mapping with an elder and an animal health worker, and historical profiles with an ice cream maker and a farmer; however, these six people refused to document their participation (33). While piloting these tools did not yield their intended outputs, they captured topics for discussing drivers influencing sanitation (Table 2). We also visited a leader in the commune and learned that this work was useful for the commune's upcoming proposal for the National Target Programme for Rural Development (a national policy initiative), of which sanitation was one component (34).

**Table 2:** Topic guidelines for discussion on changes in Hoang Tay Commune used to generate historical profile

Category	Subcategory
Society^a^	Culture^b^
	Community groups/activities^b^
	Communications^b^
Technology^a^>	Cell phones/Internet^b^
Economy^a^	Livelihoods^b^
	Farming^b^
	Income^b^
	Aquaculture^b^
Environments^a^	Roads^b^
	Water^b^
	Garbage^b^
	Toilets^b^
	Drainage system^b^
	Weather^b^
Demographics^b^	Population^b^
Health^b^	Common diseases^b^
	New diseases^b^
Education^b^	
Past projects/programmes/research^b^	

a From scenario planning methodology used by Shell Global Enterprise (13).

b From informal conversations during household visits during our scoping phase and used to probe participants when the broader categories were too general to discuss.

Our tools were adapted from *Scenario Planning with African Pastoralists* – *A* ‘How-To Guide’ (Table 1) (23). We conducted one two-hour focus group meeting per week for 4 weeks. The time between focus groups allowed for translation. We also conducted household visits to get feedback on participants’ understanding of the process. All participants were visited, but only two of the eight participants from the People's Council were willing to provide feedback. The focus group outputs were compiled based on working input from participants, captured on chart paper, and further clarified by the digital recordings. The recordings were transcribed and translated; the Analysis Method Framework was used to capture the major themes discussed (35). What follows is a description of scenario tool development and focus groups, along with their respective outputs. Process is emphasized, since the details and practice of integrated health approaches is often not comprehensively described in the literature (36) and our research objective was to adapt and test the tool (i.e. scenario planning).

## Results

In the first focus group, we explained the project's objective and purpose and defined the scenario concept, the steps needed to develop them, and the focal issue. The purpose was to construct scenarios describing what their commune would look like in 10 years if the sanitation situation remained unchanged and then to identify next steps. The objective was to assess the tool's suitability in addressing priority issues in this commune. The focal issue was designing a clean water and sanitation system (upgrading of the drainage system, waste collection, and treatment) with community contributions, without stressing household incomes. The former (designing a clean system) is part of the policy initiative mentioned above (34). The latter (without stressing household incomes) was identified during previous MSc work and echoed by community members during our scoping phase (36). Participants were asked to discuss what ‘environmental sanitation’ meant to them, and in turn, the focal issue was refined to address the garbage piling up in their commune.

In the first focus group meeting, participants identified and ranked sanitation drivers in their community. They generated a historical profile, but instead of recording significant events, they recorded changes in their commune and the perceived causes, which were grouped under broader categories (Table 2) (33). The concept of drivers was translated into Vietnamese as ‘causes’. The discussion then focused on drivers influencing sanitation. Focus group participants identified which drivers would be important over the next 10 years and ranked them using preference ranking (Tables 3 and 4) (33).

**Table 3:** Driving forces influencing solid waste management in 2020 in Hoang Tay Commune

Ranking	Driving forces	Directions in which driving forces could play out in the future
1	Awareness and behavioural change of individuals (health and environment – general hygiene, keeping the house clean, hand washing with soap, not throwing garbage everywhere)	High or low
2	Clear guidance from party and government	Strong or weak
3	Pollution of water resources (water in rice paddies contaminated with pesticides, household wastewater, irrigation water – Nhue River, contamination of ground water with arsenic)	High or low
4	Technological developments can be applied to get rid of industrial waste and household waste	High capability or low capability
5	Industrial development influences the number of factories	More solid waste or less solid waste
6	Access to information – Farmers lack computer skills to access information on the Internet	Increasing or decreasing
7	Developments in the commune – capability of investing in solid waste management strategies	High capability or low capability

**Table 4:** Key *themes* from each focus group and their context

Focus group	Context (major themes in bold)	Sample quotation
1	When discussing changes within their commune, one participant brought up the issue of Hoang Tay being **overwhelmed with problems**.	‘The water sources are polluted with Arsenic … with wastewater. The population … is imbalanced in terms of gender ….’
	Another participant pointed out the community's **increasing awareness** of environmental concerns and effects on community health.	‘… if they don't care, it will directly influence people's health … bring about new diseases … environmental pollution’.
	Many participants also emphasized the **importance of governance** when it comes to addressing the garbage issue.	‘If the leaders … don't directly care about the issue, nothing can be done. In the People's Council, we have no budget, so we must have contributions and coordination of the people ….’
2	The best-case scenario was called: *The environment is clean, green, and beautiful*. It showed a **positive projection of government activities**, showed wishful thinking, far from the current reality.	‘The roads in my commune are clean; the drainage system has been built spaciously. The People's Committee building is very beautiful, enough rooms for everyone’.
	The remainder of the scenario assigns **roles** for different groups in the community and describes how the ideal solution would work.	‘… the responsible group should be from the Women's Union, combined with a village health worker who can communicate how to separate your garbage, which can be composted, and things that can't, how to dispose of it. Those working in the cadastral sector are building roads and the sewage system and the health sector is managing, monitoring, and communicating this work’.
	The worst-case scenario was called: *Disaster*. It describes how their environment and food system is **making them sick**, with a direct economic impact, affecting their ability to access health services.	‘… our commune now has a lot of diseases … mainly originating from livestock, seafood, vegetables from the Nhue River and pesticides. I don't know where our food comes from …. The health of people deteriorates slowly, no one can go to work, and disease treatment expenses are high ….’
	The scenario also emphasized that **economic priorities** will overshadow health impacts.	‘People are concerned about making a profit, why think about health?’
	While discussing the future direction of drivers, participants insisted there were **no other possibilities** to their assumptions.	‘I want to use the word ‘nhan thuc’ (awareness) because when this is high, ‘y thuc’ (consciousness, and thus influencing their decisions) will follow in the same manner’. ‘There will be more guidance from the government; it's not possible that there will be less’.
3	Participants kept referring to when implementation of the Rural Development Proposal would begin, as there were **no other possibilities**.	‘… then wait until 2013 when we will have the land for a dump site’.
	Participants struggled to come up with other possibilities, assuming they could not address existing constraints (**no other possibilities**).	‘… this is a math problem with no solution’.
	After pushed to consider other perspectives (as a leader, the environmental sector, the business sector, and a farmer), there was agreement on possible alternative options.	‘… propaganda to increase consciousness of people (to keep the commune sanitary)’ ‘… advocate families to give up land (for government re-distribution) in order to have a place for a dumpsite’ ‘… if they still litter … one warning, a second warning, the third time is a penalty. With this in writing, then people will remember’. ‘Families (should) destroy their own (garbage)’, ‘first by separating garbage, recycling, re-using’, ‘applying (organic waste) to the fields’, ‘bury the rest’.
	Participants expressed that the first step was **someone else's responsibility**; various stakeholders are needed but the way forward is still top down (**importance of governance**).	‘The government leaders should assign tasks to stakeholders and other sectors should coordinate together, but first we have to communicate with and persuade the people’.
4	Participants were beginning to understand and learn from the process because they could articulate the reasons for each step.	‘We put the drivers in a graph because it's easier to understand, to create the best and worst cases’.
	The tool was useful for planning because it had **logical** steps.	‘This method is scientific’.
	It offered an **alternative way** of doing things (consideration of the worst case scenario) and a tool that could be **applied in other sectors**.	‘If we just have one way, we just evaluate how this will turn out. If this doesn't turn out the way we expect, this thinking is not useful’. ‘… for development of trade services, small scale industries at the provincial level, and social, cultural, and economic issues in rural areas’
	Participant feedback revealed how they were originally viewing their own participation.	‘… we answered your questions so you could do your job … write reports about our sanitation situation. But actually, we can see now that this method could be useful for us … for planning in other sectors’.

**Fig. 2:**
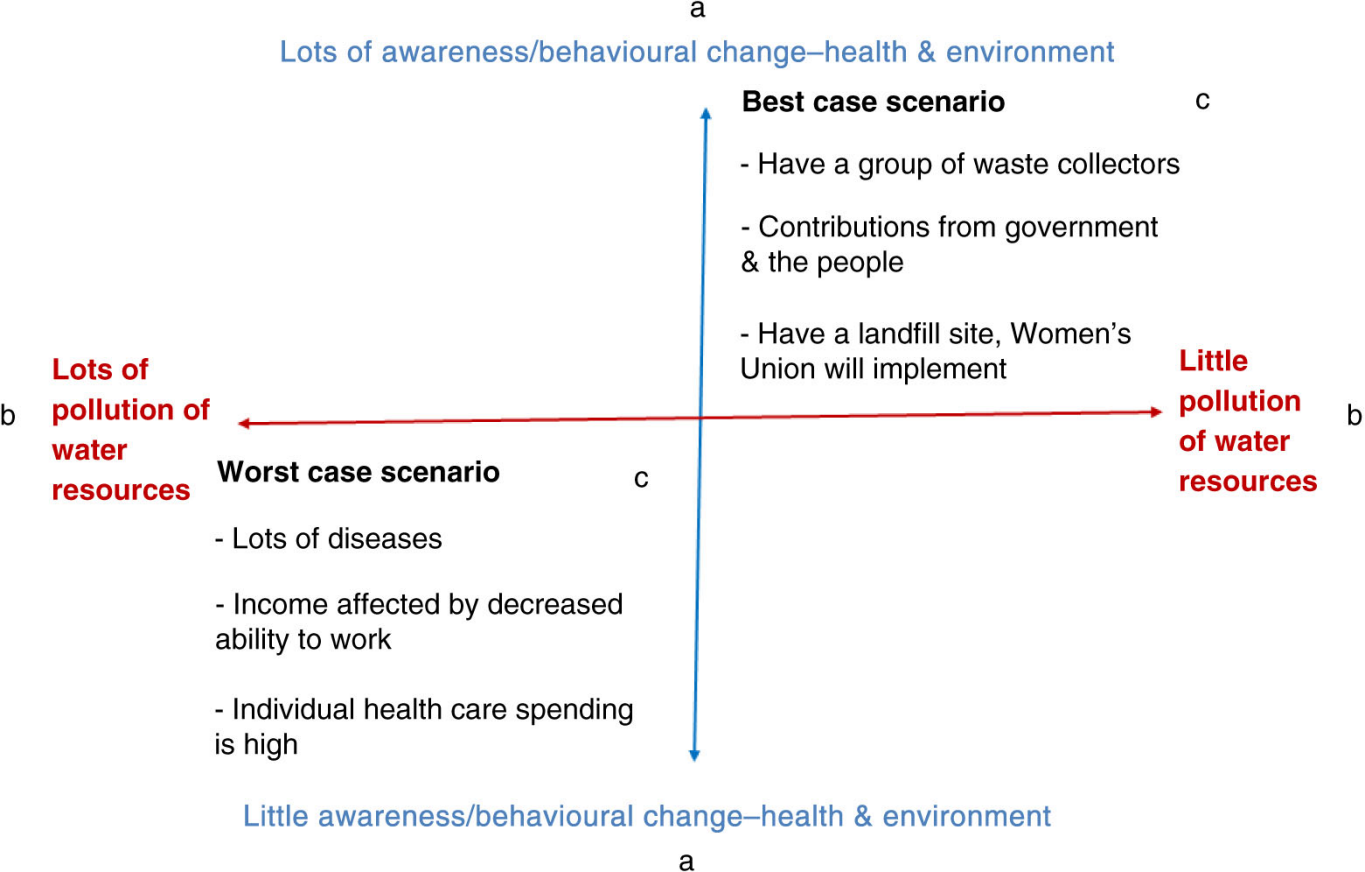
An example of a matrix showing (a) the two possible directions of driving force 1, (b) the possible directions of driving force 2, (c) each quadrant containing one outcome, a paired combination of two drivers, that was used as a basis for constructing scenarios.

In the second focus group meeting, participants identified the direction of the driving forces in the future and used these to construct scenarios. The driving forces were broad and were clarified using the digital recordings. We presented matrices, each containing the top three driving forces; each paired combination yielded four outcomes (Fig. 2). There were three possible paired combinations and we constructed scenarios for the pair identified as ‘most uncertain’. There were actually seven drivers, yielding 21 paired combinations, but we only used the top three to help participants focus the discussion. Uncertainty ranking was done by identifying the paired combination for which it was the most difficult to imagine the future. Each quadrant of the matrix was filled with options and possibilities for the four possible outcomes and formed the starting point for constructing scenarios. Our household visits revealed that it was easier to talk about things that participants could see and that were related to their everyday lives. Thus, we asked questions about the past, present, and future (if things continued to develop in this way). Guiding questions identified options, described interventions and responsible personnel, required resources and sources, deadlines, and anticipated problems. Participants were asked to imagine what their lives would look like and to share this like a story to an old friend. Due to time constraints, the outputs were limited to the best and worst case scenarios. The scenarios were constructed following this focus group in reference to quotations from the digital recordings in order to stay true to how the participants tell stories (Table 4).

**Fig. 3:**
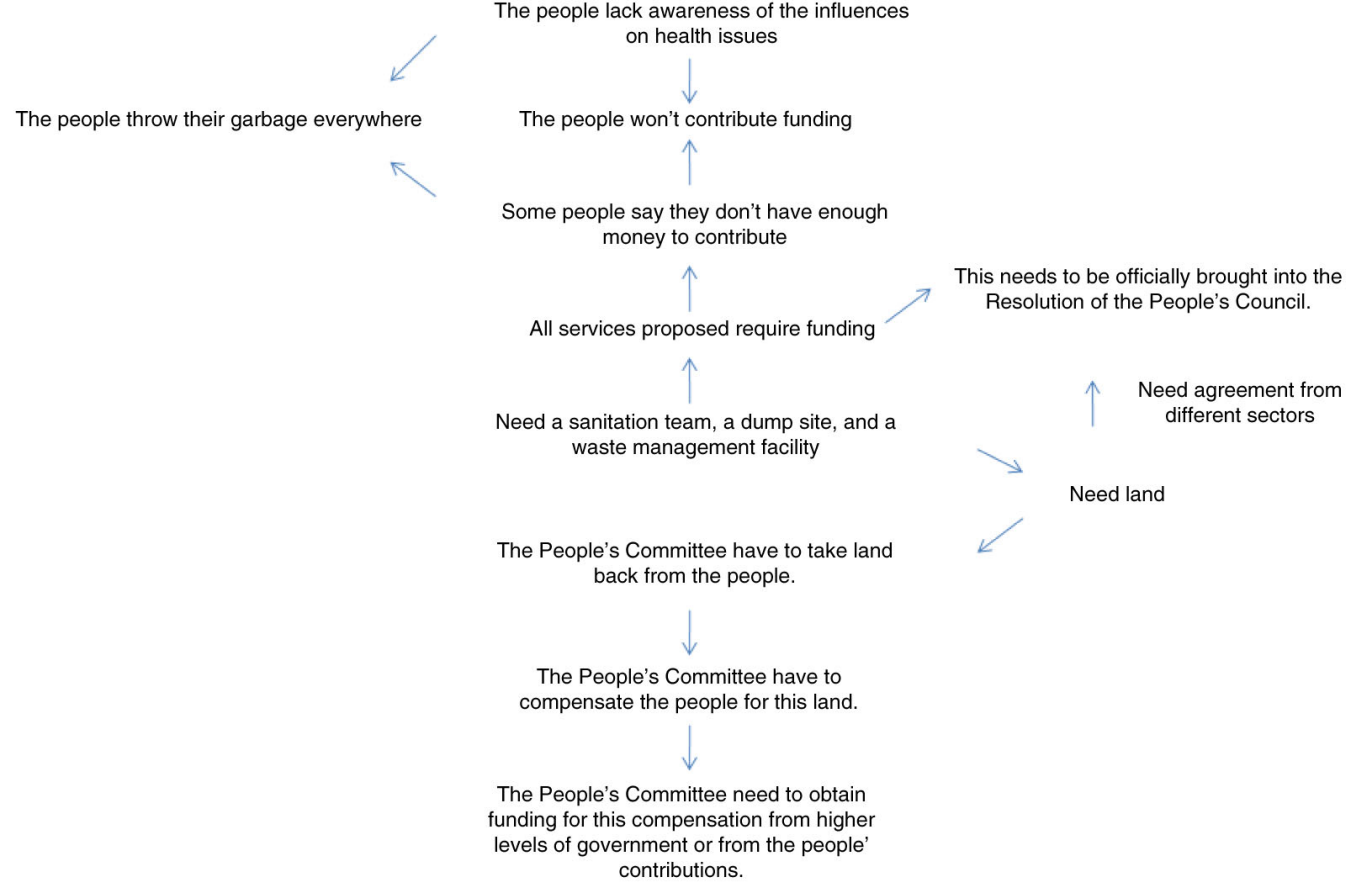
Issues and constraints to achieving the best-case scenario.

In the third focus group meeting, participants discussed the implications of the scenarios and possible options and next steps. The research assistant read the scenarios aloud and asked participants to brainstorm implications all together. Before the focus group meeting, we constructed an initial mind map of the issues and constraints of the garbage issue and their connections, based on the digital recordings (Fig. 3). Focus group participants had expressed the need for funding, government approval and guidance, and a dump site. Additions, deletions, and changes to the mind map were made together. Discussions then focused on options and next steps, given these issues and constraints, and other possible paths forward, from the perspective of a leader, the environmental sector, the business sector, and a farmer.

The discussion of options and next steps yielded a repetition of many of the issues presented as constraints (Fig. 3 and Table 4). To get the best-case scenario, many participants echoed that it was absolutely necessary to have ‘guidance from the government, official plans for a dump site, and funding for sanitation services’ as well as ‘… a sanitation team … a dump site … waste management facilities’. We had anticipated that the major output of this focus group meeting would be a specific strategy to address the garbage issue, one detailed enough to pilot. Thus, when discussions became repetitive, we steered participants towards identifying alternative options.

In the fourth focus group meeting, participants discussed their understanding of the process. We reviewed the steps and outputs and observed that most participants had taken notes from previous focus group meetings, referring to them when asked to recap the steps. Discussion then focused on the overall scenario planning process: identifying reasons for each step, what kinds of issues this could be used for, lessons learned, prior expectations, suggestions, and usefulness (Table 4).

## Discussion

As our research objective was to adapt scenario planning, this discussion focuses on the implications of our results on the tool, rather than the sanitation problem. The major finding was the description of the process of tool development and engagement with participants, which may be useful for others interested in the practice of integrated health approaches. The output of this process was the identification of options that are actionable. The self-assessment stage demonstrated participant learning – constructing a worst case scenario led participants to think about a different way of planning. It also showed a change in thinking about research participation, as active participants with two-way benefits rather than sources of data. A review focusing on the use of scenarios in forest communities includes experiences and lessons from the central provinces of Vietnam (22). It highlighted that the process helps communities reflect on their needs, vulnerabilities, and how to be more forward-thinking by developing their organizational and planning capacities. In his discussion of whether scenarios are worth the effort, Molitor *et al*. (11) has stated that one of the benefits of scenarios, as strategic conversations, is learning, which is as important as being a problem-solving tool itself. An early pioneer of scenario planning has emphasized that ‘planning means changing minds, not making plans’ (37). In other words, outcomes depend on learning – the ability of decisions makers to understand the state of the environment and to appropriately respond through action. This is the process through which participants change a shared mental model of their institution, environments, and actors. Most importantly, this learning refers to learning of those who hold power and can act on it (37).

Given this purpose, the scoping phase revealed that participant selection should be viewed as choosing boundary partners – people or groups that a project works with directly and thus have the potential to influence (38). Members from the People's Council were chosen strategically because, collectively, they had the power to act; their role is to supervise the People's Committee (local government) (39). They had administrative powers at the commune level, which are limited by the policies and regulations set by the central government. This was the case for land use, which is relevant to our focal issue. Legally, the People's Committee (at all levels of government) has the authority to makes plans and policy decisions. However, there are practical issues related to planning and management that stem from the process of decentralization of the administrative system still being top down in the delegation of tasks from central to the lower levels. Also, all the decisions and plans are dependent on the availability of allocated funding or that which can be mobilized for these decisions (39). Our engagement with them led to identification of a possible channel for scenario usage – a policy initiative for rural development, which included sanitation (34). While a large organization may have the funding and infrastructure to support scenario planning activities, these things are not (or little is) available within the community beyond project funding cycles. Thus, the potential for the application of our research findings depends on such a channel.

In our first focus group meeting, a refinement of the focal issue involved a negotiation between the research and community priorities. Initially, the wastewater drainage system was the focal issue, as it was part of the policy initiative; however, sanitation discussions always reverted to the garbage issue. This was also the case during scoping. Sanitation was too complex to talk about and community members were overwhelmed by the scale of the problem, while the garbage issue evoked very vivid images of a pressing issue from within their community, making it easier to discuss. While solid waste management was not our intended area of focus, we realized the topic needed to focus on an issue that the community already wanted to act on and felt they had some likely control over.

In the last focus group meeting, discussions of how participants viewed their own involvement showed that the benefits of participation were initially perceived to be one-way and they were conveying messages, not necessarily data. This was also consistent with what participants had told us during household visits about how and why the options that were identified based on scenarios constructed collectively would not work. Furthermore, how they viewed the researcher's role, their experiences of failure of numerous past sanitation projects/interventions, and their current positions of power and responsibility affected how they participated in later projects. In other words, this influenced how they engaged; the type of information and hence, the data they provided. Thus, awareness of their conveyed messages is an important issue in communities that have a long-standing history of being ‘researched’.

Conveyed messages and the focus group themes reinforced the status quo, while the household visits confirmed that participants were aware of this. This reinforces the need to re-examine the scenarios, as they hold the key to changing mental models and identifying other options. While the best-case scenario was a dominant narrative based on socially desirable responses, the worst-case scenario made it easier to question the limitations for achieving the best case. In the end, identified alternatives were based on considering other perspectives. In retrospect, it was difficult to break down conventional thinking without a specific strategy to promote this. It was only after the research that we learned about causal layered analysis as a tool to do this and thus increase strategic options (40). However, this was beyond the scope of our research and would require in-depth understanding and experience with it.

Without a tool to break down conventional thinking, how does one begin to change mental models? In terms of the focal issue and the scenario tool itself, understanding what facilitates learning is one starting point. In our case, this meant understanding how transdisciplinarity – namely knowledge integration (across sectors and stakeholders) – actually happens. This could be visualized using the matrix that shows a paired combination of two drivers (Fig. 2). This informs *what* was integrated and was identified from knowledge and people from different sectors. Each paired combination yields four possible outcomes, represented in each quadrant of the matrix, and each outcome forms the basis from which scenarios are constructed. This informs *how* integration could happen. The output is a narrative, but it is the tool that permits this and the engagement with participants that facilitated both our and their understanding of the process.

Our major limitation was that the study was context-specific. The data collected was influenced by the history of research in the community; community engagement was informed by *our* understanding of the local context. This was built on years of experience between our partner programme and the community and required adapting our approach to real-time feedback. Another limitation was the time constraints we faced. Scenario processes require a lot of time and it has been reported that, alone, constructing scenarios themselves have seldom been used to successfully design strategies to achieve envisioned goals (11). They are usually a part of a larger process to generate alternative futures.

Two lessons from our research that may be applicable to other contexts lies in what gave meaning to our data and helped articulate process, in order to use the data to extract lessons about the tool. The first was focusing on linkages (within policy initiatives, mandates, research or programme objectives, and community priorities) at the conceptualization and design phase of research. This was what helped us identify a channel for research applications and negotiate between research and community priorities. The second was a meta-abstraction of process during and after data collection. ‘In epistemology, the prefix meta- is used to mean about (*its own category*)’, and thus, metadata is data about the data, or in our case, describing the context of data (41). Abstraction is the process that can yield metadata and involves using only the relevant information to describe content or phenomenon. This came from our observations and questioning of data during household visits to help understand the information obtained during formal data collection.

Our focus on process is what yielded insights into the bigger challenges that this research set out to address. Building on previous studies, the conceptualization phase was an attempt to simultaneously address the questions of: 1) How to practically implement ecohealth concepts (namely, transdisciplinarity and systems thinking)? 2) How to use these concepts to address emerging/re-emerging infectious diseases?, and 3) How to communicate our research insights for applications in other contexts? It was a combination of these questions, faced with the local realities of a sanitation problem, and the desire to address the issue systemically that led to questions of how to develop community capacity and address the sustainability of research/interventions beyond project funding cycles.

In Asia, disease emergence has been commonly linked to unsafe water/sanitation and solid waste management under crowded conditions; thus, sanitation was chosen as our entry point (42–45). Whether this is viewed as a local sanitation problem or as a factor driving the global health issue of emerging infectious diseases depends on the framing of the issue and the scale at which researchers choose to work at. Thus, ecohealth could be viewed as the framing of problems; wherein the research questions shape the potential responses. The question of how to develop community capacity led us to search for an action–research tool to address the complexity of the issue, but was also cross-cutting. Other examples from the literature have addressed disease and broader health issues by examining these relationships within their ecosystem context (8). Waltner-Toews accomplished this through riparian restoration and waste management and using issues and influence diagrams to identify these relationships, instead of focusing on the parasite–gastrointestinal disease relationship (46). Hagemeijer *et al*. examined factors leading to the emergence and transmission of avian influenza H5N1 and proposed wetland restoration for wild birds to reduce their contact with domestic bird populations (47). Berbés-Blázquez used photovoice to identify a critical role of ecosystem regulating services as protection against flooding, which may result in water-borne, vector-borne, and rodent-borne disease risks (48). The major insight that could be gained from these studies and trying to answer our bigger questions was that there is no best practice for ecohealth and that the way forward lies in the development of tools that are appropriate for the context and articulating process to help clarify how to move from concept to practice.

## Conclusion

As a powerful communication tool that can be used to convene people to discuss a shared issue, scenario planning represented an ideal starting point to identify problem-solving options. Change started with the construction of scenarios and identification of options. Understanding the context of the data (the outputs of the focus groups) required engagement with the participants outside of formal data collection, namely through one month of scoping and household visits in between the focus group meetings. Engagement in this process for both the researchers and the participants led to a shift in thinking about the issue and the tool itself. This provided the space for understanding the context of data or conveyed messages. Further work should explore how to unpack the stories with the participants to increase strategic options by adapting causal layered analysis.

## References

[1] WHO (2013). Progress on sanitation and drinking-water – 2013 update.

[2] WHO (2010). UN-Water global annual assessment of sanitation and drinking-water (GLAAS) 2010: targeting resources for better results.

[3] SIWI (2004). Securing sanitation – the compelling case to address the crisis.

[4] WB (2008). Economic impacts of sanitation in Vietnam: a five country study conducted in Cambodia, Indonesia, Lao PDR, the Phillipines, and Vietnam under the Economics of Sanitation Initiative (ESI).

[5] Arya N, Howard J, Isaacs S, Mcallister ML, Murphy S, Rapport D (2009). Time for an ecosystem approach to public health? Lessons from two infectious disease outbreaks in Canada. Glob Public Health.

[6] Charron D (2012). Ecosystem approaches to health for a global sustainability agenda. EcoHealth.

[7] Wilcox B, Aguirre AA, Daszak P, Horwitz P, Martens P, Parkes M (2004). EcoHealth: a transdisciplinary imperative for a sustainable future. EcoHealth.

[8] Bunch MJ, Morrison KE, Parkes MW, Venema HD (2011). Promoting health and well-being by managing for social–ecological resilience: the potential of integrating ecohealth and water resources management approaches. Ecol Soc.

[9] Ringland G (2002). Scenarios in public policy.

[10] Senge PM, Kleiner A, Roberts C, Ross RB, Smith BJ (1994). The fifth discipline fieldbook: strategies and tools for building a learning organization.

[11] Molitor GTT (2009). Scenarios: worth the effort?. J Futures Stud.

[12] Ringland G (1998). Scenario planning.

[13] Schwartz P (1996). The art of the long view.

[14] List D (2005). Scenario network mapping: the development of a methodology for social inquiry. PhD.

[15] Shell, Scenarios: an explorer's guide (2003). Global Business Environment, Shell International. http://s05.static-shell.com/content/dam/shell/static/public/downloads/brochures/corporate-pkg/scenarios/explorers-guide.pdf.

[16] Venable JM, Li Q, Ginter PM, Duncan WJ (1993). The use of scenario analysis in local public health departments: alternative futures for strategic planning. Public Health Rep.

[17] Bierbooms J, Bongers I, van Oers H (2011). A scenario analysis of the future residential requirements for people with mental health problems in Eindhoven. BMC Med Inform Decis Mak.

[18] van Genugten MLL, Heijnen MA, Jager JC (2003). Pandemic influenza and healthcare demand in the Netherlands: scenario analysis. Emerg Infect Dis.

[19] PHAC (2011). Children and physical acitivity scenarios project – evidence-based visions of the future: executive summary.

[20] Enfors EI, Gordon LJ, Peterson GD, Bossio D (2008). Making investments in dryland development work: participatory scenario planning in the Makanya Catchment, Tanzania. Ecol Soc.

[21] Peterson GD, Beard TD, Beisner BE, Bennett EM, Carpenter SR, Cumming GS (2003). Assessing future ecosystem services: a case study of the Northern Highlands Lake District, Wisconsin. Conserv Ecol.

[22] Evans K, Jong WD, Cronkleton P (2008). Future scenarios as a tool for collaboration in forest communities. SAPIENS.

[23] Cavanna S, Abkula D (2009). Scenario planning with African pastoralists: a ‘how to’ guide.

[24] Rawluk A, Godber A (2011). Widening the scope of scenario planning in small communities: a case study use of an alternative method. Ecol Soc.

[25] GSO (2009). Completed results. The 2009 Vietnam population and housing census.

[26] CPC (2008). Annual health statistics in Hoang Tay Commune.

[27] DOH (2006). Health and environmental sanitation situation in Nhat Tan and Hoang Tay Communes.

[28] MOH (2005). Regarding issuing of the sector standards. Hygiene standards of various types of latrines. Decision of the Ministry of Health (MOH) Report.

[29] CPC (2008). Annual health statistics in Nhat Tan Commune.

[30] MONRE, ICEM (2007). Improving water quality in the day/Nhue River Basin: capacity building and pollution sources inventory.

[31] NCCR N-S (2009). Research partnerships for sustainable development in Southeast Asia: highlights of the National Centre for Competence in Research North-South (NCCR North-South) Program in Southeast Asia, 2005–2009.

[32] Nguyen-Viet H, Zinsstag J, Schertenleib R, Zurbrügg C, Obrist B, Montangero A (2009). Improving environmental sanitation, health, and well-being: a conceptual framework for integral interventions. EcoHealth.

[33] Rifkin SB, Pridmore P (2001). Partners in planning: information, participation and empowerment.

[34] Nguyen DT, Prime Minister (2010). Decision: approval of the National Target Program for Rural Development 2010–2020.

[35] Spencer L, Ritchie J, O'Connor W, Ritchie J, Lewis J (2003). Analysis: practices, principles, and processes. Qualitative research practice: a guide for social science researchers.

[36] Nguyen V (2011). Understanding the concept and practice of ecosystem approaches to health within the context of public health.

[37] De Geus AP (1988). Planning as learning. Harvard Bus Rev.

[38] Earl S, Carden F, Smutylo T (2001). Outcome mapping: building learning and reflection into development programs.

[39] Fforde A (2003). Decentralisation in Vietnam – working effectively at the provincial and local government level – a comparative analysis of Long An and Quang Ngai Provinces. http://aid.dfat.gov.au/Publications/Documents/decentralisation_vietnam.pdf.

[40] Inayatullah S (2004). Causal layered analysis: theory, historical context, and case studies.

[41] Anonymous Meta. http://en.wikipedia.org/wiki/Meta.

[42] Arunachalam N, Tana S, Espino F, Kittayapong P, Abeyewickreme W, Wai KT (2010). Eco-bio-social determinants of dengue vector breeding: a multicountry study in urban and periurban Asia. Bull World Health Organ.

[43] Sims LD, Domenech J, Benigno C, Kahn S, Kamata A, Lubroth J (2005). Origin and evolution of highly pathogenic H5N1 avian influenza in Asia. Vet Rec.

[44] WHO (2013). Water-related diseases.

[45] Zhou X, Bergquist R, Leonardo L, Yang G, Yang K, Sudomo M (2010). Chapter 6 – Schistosomiasis Japonica: control and research needs. Adv Paraitol.

[46] Waltner-Toews D (2004). Ecosystem sustainability and health: a practical approach.

[47] Hagemeijer W, Martin V, Karesh W, Newman S, Ounsted M, Madgwick J Managing wetlands for people and nature to minimize the risks of disease – an example of avian influenza. Healthy wetlands, healthy people: report of the Shaoxing City symposium.

[48] Berbés-Blázquez M (2012). A participatory assessment of ecosystem services and human wellbeing in rural Costa Rica using photo-voice. Environ Manage.

